# Methoden zur Erfassung der Krankheitsaktivität der Polymyalgia rheumatica

**DOI:** 10.1007/s00393-023-01358-x

**Published:** 2023-05-15

**Authors:** Myriam Reisch, Christian Dejaco

**Affiliations:** 1 Abteilung für Rheumatologie und Immunologie, Medizinische Universität Graz, Graz, Österreich; 2 Rheumatologie, Krankenhaus Bruneck, Südtiroler Sanitätsbetrieb: Azienda Sanitaria dell’Alto Adige, Spitalstr. 11, 39031 Bruneck (BZ), Italien

**Keywords:** Rezidiv, Biomarker, Bildgebung, Remission, Therapieansprechen, Recurrence, Biomarkers, Imaging, Remission, Response to treatment

## Abstract

Die Polymyalgia rheumatica (rPMR) ist die zweithäufigste entzündlich rheumatische Erkrankung im höheren Lebensalter. In klinischen Studien werden häufig die Remission und das Rezidiv als Endpunkte festgesetzt, jedoch existieren für diese Zustände noch keine einheitlichen Definitionen, was die Vergleichbarkeit von Studien erschwert. Der PMR-AS (PMR-Aktivitätsscore) ist derzeit der einzige für die PMR entwickelte Composite Score, durch den neben der Remission auch eine niedrige, mittlere und hohe Krankheitsaktivität definiert werden. In neueren Studien wird der PMR-AS häufig verwendet und die niedrige Krankheitsaktivität als Endpunkt festgelegt. Eine Limitation des PMR-AS ist die mögliche Beeinflussung der einzelnen Variablen durch Komorbiditäten. Beim Einsatz von Medikamenten, welche die Interleukin-6-Achse beeinflussen, sind das C‑reaktive Protein (CRP) und die Blutsenkungsgeschwindigkeit (BSG) für die Beurteilung der Krankheitsaktivität der PMR nur eingeschränkt verwertbar. Vielversprechende alternative Biomarker sind Calprotectin und Osteopontin, die bereits bei der rheumatoiden Arthritis die Erkrankungsaktivität unabhängig vom CRP widerspiegeln konnten. Darüber hinaus könnten bildgebende Verfahren wie die Sonographie, Magnetresonanztomographie und FDG(Fluordesoxyglucose)-Positronenemissionstomographie zum Monitoring der Krankheitsaktivität eingesetzt werden, wobei diese erst in weiteren Studien validiert werden müssen. Die PMR-IS (PMR-Impact Scale) ist ein Composite Score zur Erfassung der Auswirkungen von PMR auf die Patient:innen. Sie wurde allerdings bisher noch nicht in klinischen Studien angewendet. Die Entwicklung von weiteren PROs („patient reported outcomes“) für die PMR und die Definition von einheitlichen Kriterien zur Erfassung der Remission und des Rezidivs sind für die PMR wichtige zukünftige Forschungsfragen.

## Abstract (en)

Polymyalgia rheumatica (PMR) is the second most frequent inflammatory rheumatic disease in old age. Remission and recurrence are frequently used as endpoints in clinical trials; however, there is as yet no international consensus on the definition of these states, which limits the comparability of published studies. The PMR activity score (PMR-AS) is the only composite score specifically developed for PMR, which together with remission is used to define low, middle and high disease activity. In recent studies the PMR-AS was often used and low disease activity was established as endpoint. The most important limitation of the PMR-AS is the potential influence of the individual variables by comorbidities. The value of C‑reactive protein (CRP) and the erythrocyte sedimentation rate (ESR) are of restricted value in studies using drugs that influence the interleukin 6 (IL-6) axis. In these cases, calprotectin and osteopontin are promising alternative biomarkers, as they have already been shown to reflect disease activity independently of CRP in rheumatoid arthritis. Furthermore, imaging modalities including sonography, magnetic resonance imaging and fluorodeoxyglucose (FDG) positron emission tomography could also be helpful in monitoring disease activity; however, these techniques must first be validated in further studies. The PMR impact scale (PMR-IS) is a composite score to assess the impact of PMR on the patients; however, it has not yet been used in clinical studies. The development of additional patient reported outcomes (PRO) for PMR and the definition of standardized criteria for documentation of remission and recurrence are important questions in the future research agenda for PMR.

Die Polymyalgia rheumatica (PMR) ist die zweithäufigste entzündlich rheumatische Erkrankung im höheren Lebensalter nach der rheumatoiden Arthritis (RA). Die PMR manifestiert sich typischerweise ab einem Alter von 50 Jahren mit einer Spitzeninzidenz in der achten Lebensdekade. Die jährliche Inzidenz liegt zwischen 12 und 60 Fällen pro 100.000 Einwohner:innen, wobei die Erkrankung am häufigsten in Nordeuropa auftritt. Frauen sind 3‑mal so oft betroffen wie Männer. Die genaue Ätiologie für die Entstehung der Erkrankung ist bis dato noch nicht geklärt, allerdings konnte gezeigt werden, dass Polymorphismen im HLA-Klasse-II-Gen sowie genetische Varianten von Adhäsionsmolekülen und Chemokinen mit der Erkrankung assoziiert sind. Zudem wurden vermehrt Hinweise gefunden, dass bestimmte Infektionen die Erkrankung auslösen könnten, jedoch ließ sich diese Hypothese noch nicht endgültig bestätigen. Die PMR ist eng mit der Riesenzellarteriitis assoziiert und wird von vielen als ihre Minorvariante angesehen [1].

Fast alle Patient:innen mit PMR berichten über Schmerzen in den Schultern (70–95 %), im Nacken und/oder Beckengürtel (50–70 %), die von einer ausgeprägten Morgensteifigkeit begleitet werden. Etwa die Hälfte der Betroffenen weist zudem periphere Manifestationen in Form einer nichterosiven asymmetrischen Arthritis auf, die v. a. die Knie- und/oder die Handgelenke betrifft. Unspezifische Symptome wie Fatigue, Gewichtsverlust, subfebrile Temperatur, Schwäche oder Depression kommen bei etwa einem Drittel der Patient:innen vor [2, 3].

Im Verlauf der Erkrankung werden 16–21 % der Patient:innen, die ursprünglich als PMR klassifiziert wurden, mit einer Riesenzellarteriitis (RZA) diagnostiziert. Vor allem bei Betroffenen, die hohe Glukokortikoid(GK)-Dosen zur Symptomkontrolle benötigen sowie zahlreiche Rezidive erleiden und/oder bereits initial ungenügend auf die Therapie ansprechen, sollte an eine RZA gedacht werden. Eine Bildgebung (z. B. Ultraschall oder PET[Positronenemissionstomographie]-CT [Computertomographie]) der Gefäße oder alternativ eine Temporalarterienbiopsie kann in diesen Fällen zur Klärung der Diagnose beitragen. Beide Erkrankungen sind eng miteinander vergesellschaftet; 40–60 % der Patient:innen mit einer diagnostizierten RZA können auch Symptome einer PMR vorweisen [4].

Die Therapie der Wahl für die Behandlung der PMR ist die orale Gabe von GK, beginnend mit 12,5–25 mg Prednisolon-Äquivalent pro Tag, gefolgt von einer graduellen Reduktion der Dosis bis zum Absetzen. Etwa die Hälfte der Patient:innen erleidet beim Ausschleichen der GK zumindest 1‑mal ein Rezidiv. Jedes Wiederauftreten der Symptome verlängert die Dauer der Behandlung und erhöht damit die kumulative GK-Dosis. Die ACR(American College of Rheumatology)/EULAR(European League Against Rheumatism)-Leitlinien von 2015 empfehlen bei erhöhtem Rezidivrisiko, Nebenwirkungen der GK-Therapie und/oder Komorbiditäten die frühzeitige Einleitung einer Therapie mit Methotrexat zusätzlich zu GK. Randomisierte Studien zeigen, dass durch die Gabe von Methotrexat sowohl eine Verringerung des Rezidivrisikos als auch der kumulativen GK-Dosis erreicht werden kann [5].

Weitere GK-sparende Therapien werden für die PMR derzeit untersucht. Zwei rezente Studien konnten zeigen, dass Tocilizumab (TCZ), ein monoklonaler Antikörper gegen den Interleukin-6-Rezeptor sowohl bei Patient:innen, bei denen die GK-Therapie nicht ausreichend anspricht, als auch bei Neupatient:innen wirksam ist [6, 7].

## Messung der Krankheitsaktivität bei Polymyalgia-rheumatica-Patient:innen

Die Messung der Krankheitsaktivität bei Patient:innen mit PMR ist sowohl für die Beurteilung der Wirksamkeit von Medikamenten in klinischen Studien, als auch zur Therapiesteuerung in der klinischen Praxis von großer Bedeutung. Aktuell ist kein einheitlicher internationaler Konsensus zur Bestimmung der Krankheitsaktivität, ebenso wenig wie eine einheitliche Definition für Therapieansprechen, Remission und Rezidiv vorhanden. Die unterschiedlichen Kriterien sowohl in klinischen Studien als auch in der Praxis führen zu einer eingeschränkten Vergleichbarkeit der verfügbaren Daten (Tab. 1).

**Table 1:** 

Studien-ID	Anzahl der Patient:innen	Weiblicher Anteil (%)	Dauer	Intervention	Kontrollgruppe	Primärer Studienendpunkt	Sekundäre Studienendpunkte	Reklassifikation während der Studie
2010 Kreiner [8]	40	–	–	Etanercept 25 mg 2‑mal/Wo	Placebo	∆PMR-AS (15.d)	BSG, TNF‑α, IL‑6, HAQ, Tramal-Dosis	1 (incipente maligne Erkrankung)
2013 Lee [9]	78	51 (65,4 %)	–	1) Prednisolon > 15 mg	1) Prednisolon ≤ 15 mg	Rezidiv: Verschlechterung oder Wiederauftreten von klinischen Symptomen zusammen mit erhöhter BSG (> 30 mm/h) sowie CRP (> 0,5 mg/dl). Im Gegensatz dazu wurde Remission in dieser Studie als Abwesenheit von klinischen Symptomen und Normalisierung von BSG und CRP bei GK-Freiheit definiert	–	0
				2) Prednisolon + HCQ	2) Prednisolon			
				3) Prednisolon + MTX	3) Prednisolon			
2015 Viapiana [10]	52	–	12 M	Prednisolon	Methylprednisolon	Remission: Normalisierung der Entzündungswerte mit klinischer Beschwerdefreiheit	Klinische Symptome, BSG, CRP, Fibrinogen, Serum-Kortison, ACTH	0
2016 Devauchelle-Pensec [11]	20	7 (35 %)	24 Wo	TCZ	–	PMR-AS ≤ 10 Pkt. (Wo 12). Ein Rezidiv wurde in dieser Studie als PMR-AS > 10 Pkt. gewertet	PMR-AS, BSG, CRP, SF-36-Fragebogen	0
2016 Lally [12]	10	5 (50 %)	15 M	TCZ + GK	GK	Rezidivfreie Remission ohne notwendige GK-Therapie (Mo 6). Ein Rezidiv wurde als das Wiederauftreten von Symptomen von PMR, zusammen mit erhöhter BSG und/oder erhöhtem CRP definiert	Dauer der GK-Therapie, kumulative GK-Therapie, Anzahl der Rezidive	0
2021 Bonelli [6]	36	53 %	–	TCZ + GK	Placebo + GK	GK-freie Remission (Wo 16) Remission wurde definiert als Abwesenheit von Steifigkeit und Schmerzen in Schulter und/oder Hüfte. Rezidiv wurde definiert als Rückkehr von Schmerzen und Steifigkeit in Schultern und/oder Hüfte	GK-freie Remission (Wo 12 und 24), Zeit bis zum ersten Rezidiv, kumulative GK-Dosis, CRP, BSG, VAS pain, Beurteilung der Krankheitsaktivität durch Patient:innen und Behandelnde, SF-36, HAQ	0
2021 Izumi [13]	227	166 (73 %)	–	TCZ + GK	MTX + GK, Placebo + GK	Mediane GK-Dosis	CRP, GK-NW, Rezidiv	0
2022 Devauchelle-Pensec [7]	101	68 (67,3 %)	–	TCZ + GK	Placebo + GK	CRP imp PMR-AS ≤ 10 Pkt. Rezidiv wurde als PMR-AS > 10 Pkt. definiert	Prednisolon ≤ 5 mg/Tag, Dosisreduktion um ≥ 10 mg Prednisolon	0
2022 Marsmann [14]	100	–	52 Wo	MTX + GK	Placebo + GK	GK-freie Remission. Die Remission wurde als PMR-AS ≤ 10 Pkt. zusammen mit keiner notwendigen GK-Therapie definiert. Im Gegensatz dazu wurde das Rezidiv als PMR-AS > 10 Pkt. bzw. als Ermessen des Behandelnden gewertet	PMR-AS, BSG, CRP, PASS, EQ5D, HAQ, PROMIS-PF, GK-NW (GTI), MTX-NW, klinische Symptome	0

*PMR-AS* PMR-Aktivitätsscore, *Wo* Wochen, *M* Monate, *BSG* Blutsenkungsgeschwindigkeit, *CRP* C-reaktives Protein, *ACTH* adrenocorticotropes Hormon, *TNF‑α* Tumornekrosefaktor alpha, *IL‑6* Interleukin‑6, *HCQ* Hydroxychloroquin, *MTX* Methotrexat, *GK* Glukokortikoid, *TCZ* Tocilizumab, *VAS* visuelle Analogskala, *pain* Schmerz, *HAQ* Health Assessment Questionnaire, *SF-36-Fragebogen* Fragebogen zur Erfassung der Lebensqualität, *PASS* Unbedenklichkeitsprüfung (Post-Authorisation Safety Study), *EQ5D* generisches Messinstrument zum Gesundheitszustand, *PROMIS-PF* Patient-reported Outcomes Measurement Information System Physical Function, *GTI* Glukokortikoid-Toxizitätsindex (Glucocorticoid Toxicity Index), *Pkt.* Punkte, *NW* Nebenwirkungen

### Definition der Remission und des Rezidivs bei der Polymyalgia rheumatica

Die für die PMR am häufigsten verwendeten Endpunkte in klinischen Studien und in der Praxis sind das Erreichen einer Remission und/oder das Auftreten eines Rezidivs. Die Kriterien dafür wurden in den einzelnen Studien bisher aber meist unterschiedlich definiert.

In vielen Studien besteht die Gemeinsamkeit, dass die Remission als Fehlen von Symptomen der PMR und Normalisierung der Entzündungszeichen im Labor definiert wurde [15]. Dabei wird jedoch selten genauer auf die spezifischen Symptome eingegangen. Dies ist problematisch, da viele Patient:innen trotz Therapie über unspezifische Symptome wie Müdigkeit oder verminderte Leistungsfähigkeit berichten oder Komorbiditäten aufweisen, die Schmerzen und Steifegefühl verursachen können. Viele PMR-Patient:innen leiden an Arthrosen oder anderen degenerativen Veränderungen des Schultergürtels, sodass trotz adäquater Therapie der PMR Schmerzen und Bewegungseinschränkung weiterhin bestehen bleiben [16]. Beispielsweise kann es im Rahmen einer aktivierten Omarthrose zu einer akuten Verschlechterung der Beschwerden kommen, die fälschlicherweise als Rezidiv der PMR interpretiert werden kann. Wie detailliert die Symptome in klinischen Studien erhoben und der PMR bzw. etwaigen Komorbiditäten zugeordnet wurden, ist aufgrund der fehlenden Beschreibung meist nicht ausreichend beurteilbar.

Die klinische Untersuchung spielt für die Krankheitsaktivitätsbewertung der PMR nur eine untergeordnete Rolle

Die klinische Untersuchung spielt für die Bewertung der Krankheitsaktivität der PMR im Gegensatz zur RA nur eine untergeordnete Rolle. Zwar wird die Untersuchung der Hüfte laut einem Expertenkonsensus als sinnvoll erachtet, jedoch werden der Oberarm- und Oberschenkelkompressionsschmerz sowie die Schulterbeweglichkeit als nicht ausreichend sensitiv und spezifisch erachtet, wenngleich diesbezüglich Daten aus klinischen Studien fehlen [15]. Die funktionelle Beeinträchtigung von PMR-Patient:innen im täglichen Leben (z. B. Ankleiden, Haare kämmen, Aufstehen) spielt für die Bewertung der Remission derzeit kaum eine Rolle, obwohl diese für die Patient:innen sehr belastend sein kann und nicht immer mit der entzündlichen Aktivität korreliert [17]. Infobox 1 zeigt die Ergebnisse eines Expertenkonsens hinsichtlich der Parameter, die für die Definition der Remission und des Rezidivs bei der PMR herangezogen werden könnten.

#### Infobox 1 Konsens einer internationalen Expertengruppe hinsichtlich der Parameter, die zur Definition von Remission und Rezidiv bei der Polymyalgia rheumatica (PMR) von Bedeutung sind (Delphi-Expert:innen Konsensus 2010) [15]

1. Subjektive Schmerzwahrnehmung
2. Morgensteifigkeit
3. Bewegungseinschränkung des Schultergürtels
4. Benötigte GK(Glukokortikoid)-Dosis, um die Symptome zu kontrollieren
5. Erhöhte Entzündungswerte im Blut

Die Symptomatik eines Rezidivs kann teilweise ähnlich wie bei der Erstmanifestation imponieren, dennoch kann nur eine diskrete Verschlechterung einzelner Symptome vorliegen, welche die Differenzierung zu Beschwerden anderer Genese besonders herausfordernd gestaltet. Ein Rezidiv geht üblicherweise mit einer Erhöhung der Entzündungszeichen einher, allerdings können die Entzündungsparameter bei Patient:innen mit einem Rezidiv in bis zu 11 % der Fälle unauffällig sein [18].

Zwar korreliert das C‑reaktive Protein (CRP) besser mit der Krankheitsaktivität der PMR als die Blutsenkungsgeschwindigkeit (BSG), jedoch ist ein isolierter Anstieg der Entzündungswerte ohne entsprechende Klinik unspezifisch und sollte nicht in einer Steigerung der Therapie resultieren. Anhaltend erhöhte BSG und/oder CRP-Werte ohne andere Erklärung (wie beispielsweise Infekte) sind allerdings mit einem erhöhten Risiko für ein klinisches Rezidiv assoziiert, weshalb diese Patient:innen engmaschig überwacht werden sollten [19, 20]. Mitunter kann auch eine subklinische Riesenzellarteriitis für anhaltend erhöhte Entzündungswerte bei PMR-Patient:innen ursächlich sein, sodass diese Patient:innen diesbezüglich abgeklärt werden sollten.

Eine weitere Limitation bei der Interpretation von Studien sind die unterschiedlichen Grenzwerte für normale bzw. erhöhte BSG und CRP-Werte. Zudem besteht v. a. bei Studien zu modernen Substanzen, die direkt oder indirekt die IL(Interleukin)-6-Achse beeinflussen, die Schwierigkeit, dass die BSG und das CRP unabhängig von der Erkrankungsaktivität meist vollständig normal sind. Somit können diese Parameter nicht für die Beurteilung der Erkrankungsaktivität herangezogen werden. Es werden daher alternative Marker benötigt, die nicht durch diese Therapie unmittelbar beeinflusst werden (s. Abschnitt „Labor und Biomarker“).

Auch Manifestationen anderer Erkrankungen können ein Rezidiv der PMR imitieren. Eine Riesenzellarteriitis (RZA) beispielsweise kann wiederholt zu polymyalgieformen Erscheinungen führen. Bei Patient:innen kann somit im Verlauf eine RZA diagnostiziert werden [15]. Ähnliches gilt für die Reklassifikation von PMR-Patient:innen als Elderly-onset-RA im Verlauf der Erkrankung. Caporali et al. [21] konnten nachweisen, dass bei 28 % der zunächst mit PMR diagnostizieren Patient:innen die Diagnose nach 12 Monaten revidiert werden musste. Mehr als die Hälfte dieser Patient:innen wiesen schließlich eine RA auf. Beim Großteil der bisherigen klinischen Studien wurden der Umgang mit Patient:innen, die im Laufe der Erkrankung reklassifiziert wurden, ebenso die Bemühungen, die unternommen wurden, um bei wiederholten Schüben der PMR eine mögliche alternative Erkrankung auszuschließen, nicht beschrieben (s. Tab. 1).

### Polymyalgia-rheumatica-Aktivitätsscore (PMR-AS)

Leeb und Bird [22] entwickelten 2007 den Krankheitsaktivitätsscore für die PMR (PMR-AS), analog zum SDAI (Simple Disease Activity Index) bei der RA (s. Infobox 2).

Der PMR-AS ist ein häufig verwendetes Instrument zur Messung der Krankheitsaktivität: 40 % aller Publikationen zwischen 2007 und 2014, welche die Krankheitsaktivität der PMR maßen, verwendeten den PMR-AS [23].

Der PMR-AS ist eine Zusammensetzung von 4 klinischen Parametern (Schmerz, subjektiv wahrgenommene Krankheitsaktivität, Morgensteifigkeit und Beweglichkeit des Schultergürtels [EUL – „elevation of upper limb“]) sowie dem CRP (alternativ kann auch die BSG verwendet werden). Anhand der erreichten Punktezahl wird zwischen Remission (0–1,5), niedriger (< 7), mittlerer (7–17) und hoher Krankheitsaktivität (> 17) unterschieden. Ein Rezidiv wird im PMR-AS als eine Zunahme von mehr als 6,6 Punkten definiert [24]. In anderen Studien wird ein absoluter Punktewert von ≥ 9,35 Punkten als bester Cut-off-Wert zur Definition eines Rezidivs beschrieben [25].

Die Bedeutung der Unterscheidung in eine niedrige, mittlere und hohe Erkrankungsaktivität ist derzeit noch unzureichend aufgeschlüsselt. Ebenso ungeklärt ist die Frage, welcher Schwellenwert für die (bzw. welche) Therapieintervention herangezogen werden sollte.

Der PMR-AS wird in einigen Arbeiten kontrovers diskutiert, weil einzelne Parameter wie der EUL aufgrund ihrer geringen Spezifität nur von eingeschränkter Bedeutung für die Messung der Krankheitsaktivität sind. Zudem wird nur auf die Dauer und nicht auf die Intensität der Morgensteife eingegangen. Eine weitere Schwierigkeit ist die Tatsache, dass Patient:innen meist nicht oder nur schwer zwischen Symptomen der PMR und Symptomen, die durch eine (aktivierte) Arthrose oder andere degenerative Veränderungen verursacht sind, unterscheiden können und somit bei der Erfassung der Krankheitsaktivität und Schmerzen falsch hohe Werte zustande kommen [15].

Auch Medikamente wie Tocilizumab (TCZ), die direkt den CRP- (und BSG-)Wert beeinflussen, schränken die Verwendung des PMR-AS ein. Devauchelle-Pensec et al. [26] entwickelten daher einen klinischen PMR-AS, der unabhängig vom CRP berechnet werden kann (clin-PMR-AS, Infobox 2) und sich mit einer Modifikation [CRP-imp PMR-AS = 1,12 (clin-PMR-AS) + 0,26] auf dieselben Cut-off-Werte wie des originalen PMR-AS bezieht. Zusätzliche Studien zum Vergleich der verschiedenen Versionen des PMR-AS bzw. zur weiteren Validierung der CRP-imp PMR-AS-Variante sind allerdings noch notwendig.

#### Infobox 2 PMR-AS, clin-PMR-AS und CRP-imp PMR-AS [26]

PMR-AS = CRP (mg/dl) + VAS pain (0–10) + VAS phass (0–10) + (MST(min) × 0,1) + EUL (3–0)

clin-PMR-AS = VAS pain (0–10) + VAS phass (0–10) + (MST(min) × 0,1) + EUL (3–0)

CRP-imp PMR-AS = 1,12 (clin-PMR-AS) + 0,26

Interpretation: Remission: 0 bis 1,5 Punkte; niedrige Krankheitsaktivität: < 7 Punkte; mittlere Krankheitsaktivität: 7 bis 17 Punkte; hohe Krankheitsaktivität: > 17 Punkte; Rezidiv: ∆PMR-AS ≥ 6,6 Punkte, PMR-AS ≥ 9,35 Punkte

*PMR-AS* PMR-Aktivitätsscore, *CRP* C-reaktives Protein, *VAS* visuelle Analogskala, *pain* Schmerz (0 = kein Schmerz bis 10 = nicht auszuhaltender Schmerz), *phass* Krankheitsaktivität (Beurteilung durch die Ärzt:innen), *MST* Morgensteifigkeit, *min* Minuten, *EUL* „elevation of upper limb“, Fähigkeit, die Arme zu bewegen (0 = über Schulterhöhe, 1 = bis Schulterhöhe, 2 = unterhalb Schulterhöhe, 3 = keine Elevationsbewegung möglich)

Der primäre Endpunkt bei klinischen Studien, die sich nicht auf den PMR-AS beziehen, ist die Remission. Bei Studien, die den PMR-AS (bzw. dessen Varianten) verwenden, stellt die niedrige Krankheitsaktivität das vorrangige Therapieziel dar. Hierbei stellen sich die Fragen, inwiefern eine niedrige Krankheitsaktivität für Patient:innen akzeptabel ist, welcher der für Patient:innen minimale klinisch bedeutsame Unterschied ist, welcher beim Wechsel von einer moderaten (PMR-AS 7–17) zu einer niedrigen Krankheitsaktivität (PMR-AS < 7) beobachtet wird und wie oft bei PMR-Patient:innen, die auch degenerative Veränderungen aufweisen, ein PMR-AS ≤ 1,5 überhaupt erreichbar ist [7].

### Patient:innen-basierte Outcome-Parameter zur Messung der Krankheitsaktivität

Bisher wurden nur wenige Patient:innen-basierte Outcome-Parameter (PROs) speziell für die PMR entwickelt. Für viele Aspekte der Erkrankung werden in Studien generische Instrumente wie der Health Assessment Questionnaire (HAQ) für die Erfassung der körperlichen Funktion oder der SF-36 (Fragebogen zur Erfassung der Lebensqualität) bzw. der EQ5D (generisches Messinstrument zum Gesundheitszustand) für die Messung der Lebensqualität verwendet.

Eine OMERACT(Outcome Measures in Rheumatoid Arthritis Clinical Trials)-Arbeitsgruppe, die an der Entwicklung neuer Scores arbeitet, hat für die PMR 4 essenzielle Domänen definiert, die in allen Studien erfasst werden sollten:

- Schmerzen,
- körperliche Funktion,
- Steifigkeit und
- systemische Entzündung.

Die ersten 3 dieser Domänen gehören dabei in die Kategorie der PROs [17]. Die Instrumente für deren Erfassung wurden bis dato aber nur teilweise definiert. Bezüglich der Gelenksteife beispielsweise ist nicht eindeutig geklärt, ob die Schwere oder die Dauer gemessen werden soll und ob zur Messung eine visuelle Analogskala (VAS, üblicherweise mit einem Bereich von 0–100 mm, wobei 0 dem besten und 100 dem schlechtesten Wert entspricht) oder eine numerische Ratingscala (NRS, meist in ganzen Zahlen von 0–5 mit 0 = bester und 5 = schlechtester Wert) verwendet werden soll. Zudem wird oft in den Fragebögen nicht auf das Zeitintervall eingegangen (zum Beispiel: „in der letzten Woche“, „in den letzten 24 h“). Auch Begriffe wie „Morgensteifigkeit“ und „Gelenksteifigkeit“ stellen eine Herausforderung dar. Oftmals wird nur von der Morgensteifigkeit gesprochen, obwohl von Patient:innen berichtet wird, dass die Steifigkeit der Gelenke über den gesamten Tag verteilt auftreten kann [27]. Für Patient:innen mit PMR kann auch die Differenzierung zwischen Gelenksteifigkeit und Schmerzen schwierig und die einflussfreie Erfassung Ersterer eine Herausforderung sein.

Ein zusätzlicher Aspekt ist die Erfassung von Müdigkeit, Schlafstörungen und depressiver Symptomatik, die jedoch für Patient:innen von großer Bedeutung sind. Bis dato stehen keine spezifischen Fragebögen bzw. Instrumente für Betroffene zur Verfügung [17, 28].

### PMR-Impact Scale (PMR-IS)

Ein rezent entwickeltes PMR-spezifisches PRO-Instrument ist die PMR-Impact Scale (PMR-IS). Diese ist ein Composite, die folgende Bereiche umfasst:

- klinische Symptome (Schmerz, Gelenksteifigkeit, Schwäche und Fatigue),
- körperliche Funktionseinschränkungen (z. B. Körperpflege, Ankleiden),
- emotionales und psychisches Wohlbefinden (z. B. Stimmungslage) sowie
- GK-assoziierte Nebenwirkungen.

Die PMR-IS dient zur Erfassung von PROs und der Evaluation des Einflusses dieser auf die Patient:innen, nicht aber zur Bewertung der Erkrankungsaktivität. Die PMR-IS wurde bisher nur anhand von klinischen Daten ausgewertet und nicht mit laborchemischen Parametern sowie der Bildgebung verglichen. Darüber hinaus wird nur die GK-Therapie in den Score integriert und die Einnahme anderer Medikation in der PMR-IS nicht berücksichtigt. Zusätzliche Studien zur Validierung sind notwendig, bevor die PMR-IS definitiver Bestandteil von klinischen Studien werden kann [29].

## Weitere Methoden zur Beurteilung der Krankheitsaktivität der Polymyalgia rheumatica

Aufgrund von Komorbiditäten sowie neuen Therapieoptionen, die sowohl die Laborparameter als auch die PROs beeinflussen, sind alternative Marker für eine präzise Einschätzung der Erkrankung notwendig.

### Labor und Biomarker

Neben der Erhöhung von BSG und CRP werden bei Patient:innen mit PMR mehrere unspezifische Laborveränderungen beobachtet, die mehrheitlich die systemische Entzündungsreaktion widerspiegeln und sich unter GK-Einnahme meist normalisieren: Dazu gehören eine normozytäre normochrome Anämie, eine milde Leukozytose (die durch den Einfluss der GK fortbestehen kann), eine Thrombozytose, die Vermehrung von Gammaglobulinen und eine Erhöhung zirkulierender Immunkomplexe [16].

Eine Erhöhung der Serumwerte an löslichen IL-6-Rezeptoren wurde bei Patient:innen mit aktiver PMR beobachtet und stellt einen prädiktiven Faktor für das Auftreten eines Rezidivs dar. Allerdings ist der Zusatznutzen der Bestimmung von löslichen IL-6-Rezeptoren neben dem CRP limitiert, sodass diese Methode nicht routinemäßig zur Anwendung kommt [30]. Auch bei Patient:innen, die mit TCZ behandelt werden, bringt die Messung keine zusätzlichen Informationen, weil sowohl IL‑6 als auch sIL-6-Rezeptor unter der Therapie ansteigen können, während es unter GK-Therapie zu einer Normalisierung dieser Werte kommt. Dies wurde für die RA [31] gezeigt, zur PMR gibt es diesbezüglich noch keine Daten.

Auch andere Zytokine wie TNF(Tumornekrosefaktor)-α oder IL-1-Rezeptorantagonist und Chemokine wie CXCL9 und CXCL13 sind bei Patient:innen mit aktiver PMR erhöht und sinken nach GK-Therapie ab. Allerdings konnte auch für diese bisher kein Zusatznutzen gegenüber der Bestimmung von BSG und CRP gefunden werden [32, 33].

Die Neutrophile-Lymphozyten-Ratio (NLR), der Plättchen-Lymphozyten-Quotient (PLR) sowie die Plasmaviskosität reflektieren zwar die systemische Entzündungsreaktion zum Zeitpunkt der Diagnosestellung, jedoch sind diese für die Bestimmung der Krankheitsaktivität nur wenig geeignet [34, 35].

Osteopontin (OPN) ist ein multifaktorielles Zytokin, das teils unabhängig von der IL-6-Signalkaskade ist und somit einen vielversprechenden alternativen Biomarker für Studien darstellt, in denen IL-6(‑Rezeptor)-blockierende Medikamente eingesetzt werden. OPN ist nicht nur an der Knochensubstanzbildung beteiligt, sondern wirkt chemotaktisch unter anderem auf T‑Zellen, Makrophagen und dendritische Zellen. Eine Überexpression von OPN lässt sich bei zahlreichen Autoimmunerkrankungen wie RA, systemischem Lupus erythematodes (SLE), multipler Sklerose und Diabetes mellitus Typ 1 nachweisen [37].

Osteopontin und Calprotectin sind vielversprechende alternative Biomarker

Bei RZA-Patient:innen mit initial erhöhten OPN-Werten kam es nach Therapieeinleitung zu einer signifikanten Reduktion, zudem korrelierte OPN mit der Wahrscheinlichkeit des Auftretens eines Krankheitsrezidivs. Derzeit ist noch unbekannt, inwiefern OPN-Spiegel im Blut den Schweregrad der Krankheitsaktivität darstellen [38]. Izumi et al. [39] untersuchten OPN-Werte bei Patient:innen mit RA vor Beginn einer TCZ-Therapie. Dabei wurde beobachtet, dass niedrige OPN-Level mit einer erhöhten Wahrscheinlichkeit für das Erreichen einer klinischen Remission assoziiert sind. Andererseits konnte bei Patient:innen mit RA keine Korrelation zwischen OPN und der aktuellen Krankheitsaktivität festgestellt werden [40]. Für den Stellenwert von OPN als Biomarker für das Monitoring der Krankheitsaktivität von PMR-Patient:innen sind bisher keine Studien vorhanden.

Das Plasma-Calprotectin (S100A8/A9) ist ein weiterer Laborparameter, der im Gegensatz zum CRP unabhängig von der IL-6-Achse ist und im Vergleich zu Osteopontin technisch leichter zu messen ist. Bei mehreren rheumatischen Erkrankungen wie RA, Morbus Still, ankylosierender Spondylitis und Psoriasisarthritis ist eine Korrelation zwischen den Calprotectin-Werten und der Krankheitsaktivität nachweisbar [41]. Unter Therapie mit TCZ konnte das Calprotectin bei Patient:innen mit RA im Gegensatz zum CRP die Krankheitsaktivität der RA widerspiegeln [42]. Bei RZA-Patient:innen mit hoher Krankheitsaktivität fanden sich deutlich höhere Calprotectin-Werte verglichen mit Patient:innen in Remission [43]. Für die PMR sind bisher nur wenige Daten vorhanden. Unter oraler GK-Therapie kam es auch zu einem Absinken der Calprotectin-Werte im Blut, zudem korrelierten die Serum-Calprotectin-Werte mit dem CRP, der BSG und der klinischen Krankheitsaktivität [44]. Derzeit gibt es keine Daten zur Wertigkeit von Calprotectin unter IL-6-Inhibitor-Therapie bei PMR-Patient:innen. Eine weitere Limitation des Calprotectins sind das Fehlen von international anerkannten Normwerten sowie die Tatsache, dass in den meisten Routine-Laborpanels das Calprotectin bisher nicht enthalten ist (Tab. 2).

**Table 2:** 

Laborparameter	Vergleich mit anderen Parametern zur Bestimmung der Krankheitsaktivität von PMR	Korrelation mit CRP	Assoziation zur Krankheitsaktivität	Kommentar
Serum IL‑6 und löslicher IL-6 Rezeptor [30, 31]	Klinische Symptome, CRP > 0,5 mg/dl, BSG > 30 mm/h	Ja	–	Limitierter Zusatznutzen zu CRP, somit keine Routinebestimmung Unter TCZ-Therapie Anstieg von IL‑6 und sIL-6-Rezeptor Erhöhte Werte stellen einen prädiktiven Faktor für das Auftreten eines Rezidivs dar
TNF‑α [32]	CRP, BSG, IL‑6	–	–	Kein Zusatznutzen im Vergleich zu CRP und BSG bei der Messung der Krankheitsaktivität
IL-1-Rezeptorantagonist [32]	CRP, BSG, IL‑6	–	–	Kein Zusatznutzen zu CRP und BSG Unter GK-Therapie Anstieg von IL-1-Rezeptorantagonist
Chemokine CXCL9 und CXCL13 [33]	CRP, BSG	CXCL13 korreliert mit BSG/CRP	Erhöhte CXCL13-Werte auch nach GK-Therapie (3. M) Zum Diagnosezeitpunkt Korrelation von CXCL13 mit Krankheitsaktivität	Kein Zusatznutzen zu CRP und BSG
Neutrophile-Lymphozyten-Ratio (NLR) [34]	Klinische Symptome, CRP, BSG	Ja	Wert korreliert zum Diagnosezeitpunkt mit Krankheitsaktivität. Danach zur weiteren Beurteilung nur wenig geeignet, da eine Unterscheidung zwischen Remission und chronischem Verlauf nicht möglich ist	Patienten mit zusätzlichen Fiebersymptomen hatten erhöhte Werte von NLR
Plättchen-Lymphozyten-Ratio (PLR) [34]	Klinische Symptome, CRP, BSG	Ja	Wert korreliert zum Diagnosezeitpunkt mit Krankheitsaktivität, danach zur weiteren Beurteilung nur wenig geeignet, da eine Unterscheidung zwischen Remission und chronischem Verlauf nicht möglich ist	Patienten mit Kopfschmerzsymptomen hatten erhöhte Werte von PLR
Plasmaviskosität [35, 36]	BSG	Nein	Zur Bestimmung der Krankheitsaktivität wenig geeignet, da diese keine höhere Sensitivität im Vergleich zur BSG hat	Hohe Plasmaviskosität zum Diagnosezeitpunkt korreliert mit erhöhtem Risiko für prolongierte GK-Therapie und Manifestation der RZA
Osteopontin (OPN) [37–40]	–	Ja, sowie Korrelation zu BSG	Widersprüchliche Angaben bezüglich Assoziation von OPN und aktueller Krankheitsaktivität Niedrige Level von OPN sind mit Remission assoziiert	Unabhängig vom IL-6-Signalweg Bisher ist unklar, inwiefern OPN-Level den Schweregrad der Erkrankung widerspiegeln OPN-Werte korrelieren zum Diagnosezeitpunkt mit der Wahrscheinlichkeit des Auftretens eines Rezidivs bei RA und RZA Keine Daten zu Krankheitsaktivität der PMR
Calprotectin (S100A8/A9) [41–44]	CRP, BSG	Ja, sowie Korrelation zu BSG	Signifikante Assoziation zur Krankheitsaktivität bei RA, Morbus Still, ankylosierender Spondylitis, Psoriasisarthritis, Sjögren-Syndrom, juvenile Arthritis, SLE	Unabhängig vom IL-6-Signalweg Calprotectin korreliert mit der GK-Dosis Fehlen von international anerkannten Normwerten Derzeit keine Routinebestimmung

*TNF‑α* Tumor-Nekrose-Faktor alpha, *BSG* Blutsenkungsgeschwindigkeit, *CRP* C-reaktives Protein, *IL‑6* Interleukin 6, *GK* Glukokortikoid, *TCZ* Tocilizumab, *OPN* Osteopontin, *RA* rheumatoide Arthritis, *RZA* Riesenzellarteriitis, *SLE* systemischer Lupus erythematodes

### Bildgebende Verfahren

In den letzten Jahren hat der Ultraschall (US) zunehmend an Bedeutung in der Diagnostik der PMR gewonnen. Für das Monitoring der Krankheitsaktivität gibt es hingegen erst wenige Studien. So konnten Jiménez-Palop et al. [45] nachweisen, dass es unter GK-Therapie nicht nur zu einer Regredienz der klinischen Symptomatik, sondern auch zu einem Rückgang der im Ultraschall nachweisbaren entzündlichen Veränderungen kam. Zum Diagnosezeitpunkt wurden bei 69 % der Patient:innen zumindest eine der folgenden US-Läsionen (subakromiale-subdeltoide Bursitis, Bizepssehnentenosynovitis, coxofemorale Synovitis oder glenohumerale Synovitis) nachgewiesen, nach 4 und 12 Wochen unter GK-Therapie wurden diese nur mehr bei der Hälfte der Patient:innen gefunden. Auch Machioni et al. [46] zeigten, dass es unter GK-Therapie zu einer Verbesserung der entzündlichen Läsionen kam. Zum Diagnosezeitpunkt wurden eine subakromiale und subdeltoide Bursitis bei 73,7 % rechtsseitig, bei 78,9 % linksseitig und bei 61,4 % bilateral, eine Bizepstenosynovitis des Caput longum bei 82,5 % rechtsseitig, bei 87,7 % linksseitig und bei 71,9 % bilateral und eine glenohumeralen Synovitis bei 28,1 % rechtsseitig, bei 22,8 % linksseitig und bei 15,8 % der Patient:innen bilateral sonographisch dargestellt, jedoch zeigten sich nach 24 Wochen (± 3 Wochen) eine subakromiale und subdeltoide Bursitis nur noch bei 29,8 % rechtsseitig, bei 33,3 % linksseitig und bei 19,3 % bilateral, eine Bizepstenosynovitis des Caput longum bei 45,6 % rechtsseitig, bei 35,1 % linksseitig und bei 26,3 % bilateral und eine glenohumerale Synovitis bei 5,3 % rechtsseitig, bei 1,8 % der Patient:innen jeweils linksseitig und bilateral im Ultraschall.

Unter GK-Therapie kommt es zu einem Rückgang der im Ultraschall nachweisbaren entzündlichen Veränderungen

Zu Therapiebeginn zeigten 8 von 24 Patient:innen (33,3 %) ein positives Power-Doppler-Signal im Bereich der subakromialen und subdeltoiden Bursitis. Nach 24 ± 3 Wochen unter Therapie wies lediglich nur noch 1 von 24 Patient:innen (4,2 %) ein positives Power-Doppler-Signal auf. Bei 59 % der Patient:innen wurden jedoch trotz Erreichen der klinischen Remission oder der niedrigen Krankheitsaktivität weiterhin sonographische Veränderungen gefunden, deren Bedeutung bis dato noch unklar ist.

Für die FDG-PET-CT (Fluodesoxyglukose-Positronenemissionstomographie-Computertomographie) liegen die meisten Untersuchungen zur Diagnostik vor, während nur wenige Studien die Bedeutung dieser Technik zur Erfassung der Krankheitsaktivität untersucht haben. Unter Therapie mit TCZ kam es bei Patient:innen mit PMR nach 12 Wochen zu einem Rückgang der entzündlichen Läsionen in der FDG-PET-CT in allen betroffenen Gelenken außer der Schulter und des zervikalen Processus spinosus [47]. Auch Blockmann et al. [48] konnten unter GK-Therapie nach 3 Wochen einen Rückgang der FDG-Anreicherung beobachten. In Woche 6 kam es jedoch zu keiner weiteren Verbesserung der entzündlichen Läsionen.

In der Magnetresonanztomographie (MRT) ließ sich unter GK-Therapie ein Rückgang der extrakapsulären entzündlichen Veränderungen nachweisen [49].

In Medikamentenstudien wurden bisher weder der US noch die PET-CT oder MRT für die Erfassung der Krankheitsaktivität angewendet. Für die Beurteilung der Krankheitsaktivität mittels Bildgebung existieren bisher keine einheitlichen Scoringsysteme.

## Risikofaktoren für das Auftreten eines Polymyalgia-rheumatica-Rezidivs

Neben der Messung der Erkrankungsaktivität ist deren Prognose ein wichtiges Element zur Beurteilung von Patient:innen mit PMR und könnte in Zukunft für die Stratifizierung von Patient:innen in klinischen Studien von Bedeutung sein. Risikofaktoren für eine erhöhte Rezidivrate und eine verlängerte Krankheitsdauer sind das Auftreten der PMR vor dem 60. Lebensjahr, das Vorliegen einer peripheren Arthritis, sehr hohe BSG zum Beginn der Erkrankung, eine stark gesteigerte Plasmaviskosität und stark erhöhte Serumwerte an löslichen IL-6-Rezeptoren (sIL-6R-Level > 56 ng/ml) [30].

Ein erhöhtes CRP von mehr als 2,5 mg/dl sowie persistierend erhöhte CRP-Werte auch nach Einleitung der GK-Therapie sind mit einer länger andauernden GK-Therapie verbunden, während normale CRP-Werte 1 Monat nach Therapiebeginn mit einer höheren Wahrscheinlichkeit für eine GK-freie Remission assoziiert sind [50].

Das Risiko für ein Wiederaufflammen der Erkrankung ist auch durch eine initial hohe GK-Dosis sowie eine schnelle Dosisreduktion erhöht. Im Gegensatz dazu deuten einige Studien darauf hin, dass eine niedrige Initialdosis und eine langsame Reduktion der Steroidmenge mit einer geringeren Rezidivrate assoziiert sind. Weitere Risikofaktoren für ein Rezidiv sind der Nachweis der Allele HLA-DRB1*04 und *0401 sowie der IL-6-Promotor–174 G/C-Polymorphismus und der GG241-ICAM-1-Genotyp [51]. Zudem deuten erhöhte Serumwerte von Angiopoetin‑2 zum Diagnosezeitpunkt auf einen ungünstigen Verlauf der PMR, insbesondere auf eine länger andauernde GK-Therapie, hin (Infobox 3) [52].

### Infobox 3 Risikofaktoren für einen rezidivierenden Verlauf der Polymyalgia rheumatica (PMR) [50, 51]

- Auftreten vor dem 60. Lebensjahr
- Periphere Arthritis
- Rasche Reduktion der GK-Dosis
- Initial hohe GK-Dosis
- Initial erhöhte CRP-Werte ≥ 2,5 mg/dl
- Nachweis von HLA-DRB1*04 und *0401
- Nachweis von IL-6-Promotor–174 G/C-Polymorphismus und GG241-ICAM-1-Genotyp
- Erhöhte Werte von Angiopoietin‑2, löslichen IL-6-Rezeptoren und erhöhte Plasmaviskosität

*PMR* Polymyalgia rheumatica, *GK* Glukokortikoid, *CRP* C-reaktives Protein, *BSG* Blutsenkungsgeschwindigkeit, *IL‑6* Interleukin‑6, *ICAM* interzelluläres Adhäsionsmolekül 1

## Schlussfolgerung und Ausblick

Geeignete Instrumente für die Beurteilung der Krankheitsaktivität bei Patient:innen mit PMR sind sowohl für die Behandlung in der klinischen Praxis als auch zur Beurteilung der Effektivität von neuen Medikamenten in klinischen Studien notwendig. Zwar werden die Begriffe Remission und Rezidiv als Endpunkte in zahlreichen Studien verwendet, jedoch gibt es noch keinen Konsens darüber, wie diese definiert sein sollten. Folglich ist die Vergleichbarkeit von Studien eingeschränkt. In neueren Studien wird häufig der PMR-AS verwendet, welcher der einzige für die PMR entwickelte Composite Score ist. Limitationen des PMR-AS sind die mögliche Beeinflussung des Scores durch Komorbiditäten, während eine klinische Variante des Scores dessen Verwendung auch dann möglich macht, wenn CRP und BSG nicht verwertbar sind.

Durch Medikamente, die den IL-6-Signalweg beeinflussen, können die etablierten Entzündungswerte wie das CRP und die BSG zur Erfassung der Krankheitsaktivität nicht oder nur sehr eingeschränkt verwendet werden. Alternative Biomarker wurden bereits in einigen Studien untersucht. Vielversprechend sind das Calprotectin und das Osteopontin, die beide unabhängig von der IL-6-Achse die Krankheitsaktivität widerspiegeln. Bildgebende Verfahren wie die Sonographie, FDG-PET-CT und MRT können eine Alternative zur objektiven Beurteilung der Krankheitsaktivität sein. Anhand der Bildgebung kann bei persistierend erhöhten Entzündungswerten trotz klinischer Remission oft eine (subklinisch verlaufende) RZA diagnostiziert werden. Die Bedeutung von in der Bildgebung nachgewiesenen entzündlichen Läsionen im Schulter- und/oder Beckengürtel bei Patient:innen in klinischer Remission ist bis dato noch unklar.

Derzeit gibt es noch wenige Studien zu PMR-spezifischen PROs. In aktuellen Studien werden für bestimmte Aspekte wie die körperliche Funktion (HAQ) oder Lebensqualität (SF-36, EQ5D) generische Scores verwendet. Die PMR-IS ist ein kürzlich publiziertes Instrument zur Erfassung der Bedeutung der PMR und deren Therapie für Patient:innen. Bisher wurde dieser Score allerdings noch nicht in klinischen Studien angewendet. Neben der Entwicklung von weiteren PROs ist die Definition von international einheitlichen Kriterien zur Erfassung von Remission und Rezidiv eine der wichtigsten zukünftigen Forschungsfragen für die PMR.

## Fazit für die Praxis

- Derzeit existieren keine einheitlichen Definitionen für das Therapieansprechen, die Remission und das Rezidiv der Polymyalgia rheumatica (PMR).
- Der PMR-AS (PMR-Aktivitätsscore) ist der derzeit einzig verfügbare Composite Score zur Erfassung der Krankheitsaktivität bei der PMR. Die wichtigste Limitation ist die Beeinflussung des Scores durch Komorbiditäten.
- Eine klinische Variante des PMR-AS, die angewendet werden kann, falls das C‑reaktive Protein (CRP) nicht verwertbar ist, wurde definiert.
- Calprotectin und Osteopontin stellen vielversprechende neue Biomarker dar, die unabhängig von der IL(Interleukin)-6-Signalkaskade sind.
- FDG-PET-CT (Fluodesoxyglukose-Positronenemissionstomographie-Computertomographie) und Sonographie sind ebenso vielversprechende Methoden zur Beurteilung der Krankheitsaktivität, weitere Studien sind aber notwendig.
- Die PMR-IS (PMR-Impact Scale) ist ein Composite Score zur Erfassung der Bedeutung der PMR und deren Therapie für Patient:innen.
